# Supplementary material for: Hippocampal GAD67 Transduction Using rAAV8 Regulates Epileptogenesis in EL Mice
Source: Mol Ther Methods Clin Dev. 2019 Jan 8;13:180–6. doi: 10.1016/j.omtm.2018.12.012 (PMC6369250; doi:10.1016/j.omtm.2018.12.012)
Supplement: Document S1. Figures S1–S3 [file mmc1.pdf]

OMTM, Volume *13*

Supplemental Information

Hippocampal GAD67 Transduction Using rAAV8 Regulates Epileptogenesis in EL Mice

Kuniko Shimazaki, Takashi Kobari, Keiji Oguro, Hidenori Yokota, Yuko Kasahara, Yoshiya Murashima, Eiju Watanabe, Kensuke Kawai, and Takashi Okada

Supplemental Figures and Legends

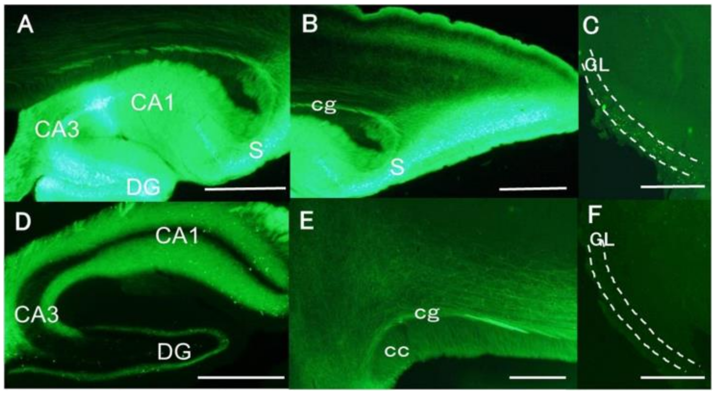

Figure S1. Anterograde and Retrograde Transduction of rAAV8-GFP.
Representative micrographs of 40-µm sagittal sections were prepared. After 8 weeks of rAAV8-GFP treatment, strong GFP expression was observed in the injection site of hippocampus (A). In the contralateral site, many GFP-positive fibers and a few GFP-positive cells were observed (D, E). A little GFP expression was seen in the ipsilateral site of olfactory bulb (C). S, subiculum; CA1, field CA1 of Ammon's horn; CA3, field CA3 of Ammon's horn; DG, dentate gyrus; cc, corpus callosum; GL, glomerular layer; cg; cingulum. Bars = 500 µm for whole sections.

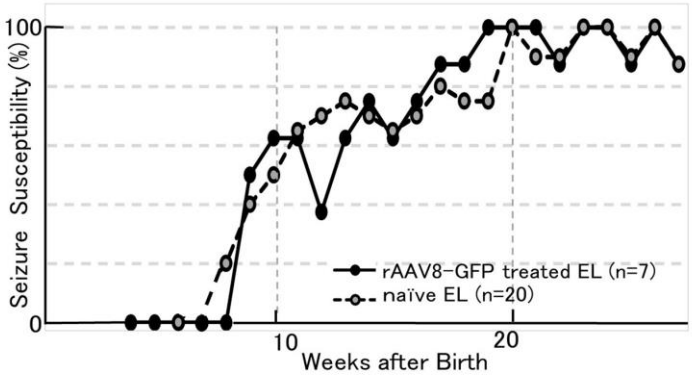

Figure S2. Development and Seizure Susceptibility of EL Mice
The seizure susceptibility increases with growth. In naïve EL mice, seizure susceptibility begins 9 weeks of age and reached about 100% around 20 weeks of age. There is no difference between naïve and rAAV8-GFP treated mice. In addition to tonic-clonic convulsion, behavioral signals of seizure activity in EL mice could include tail raising, head nodding, tail stiffening, facial/forelimb clonus and wild running. Mice that exhibited vocalization and twitching without the characteristics of behavioral signs were not considered seizure activity. A tonic-clonic convulsion is seen around 12 weeks of age.

Figure S3

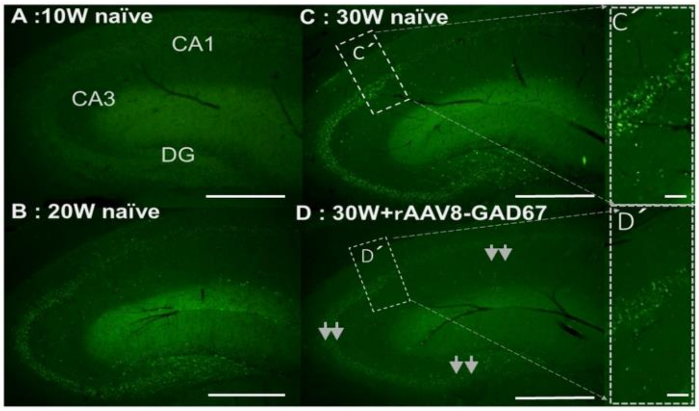

Figure S3. Representative Images of c-Fos Immunostaining in the Hippocampus.
The mice were sacrificed within 2 h after the final stimulus. For histochemical analysis, five mouse brains were fixed, and sagittal sections were prepared. Increased staining was clearly visible in the hippocampus of naïve EL mice. In 20-weeks-old naïve EL mice, c-Fos expression was upregulated in the CA3 region and DG (B). Moreover, the c-Fos expression was upregulated in the CA3 region in 30-week-old naïve EL mice (C and C´). At 21 weeks post-injection c-Fos expression levels were downregulated in 30-week-old EL mice compared to those of naïve EL mice (D and D´). Arrows indicate downregulated c-Fos expression. Scale bars indicate 250 µm and 100 µm. Double arrows indicate areas with reduced c-Fos expression.
