# Supplementary material for: Hippocampal GAD67 Transduction Using rAAV8 Regulates Epileptogenesis in EL Mice
Source: Mol Ther Methods Clin Dev. 2019 Jan 8;13:180–6. doi: 10.1016/j.omtm.2018.12.012 (PMC6369250; doi:10.1016/j.omtm.2018.12.012)
Supplement: Document S2. Article plus Supplemental Information [file mmc2.pdf]

Molecular Therapy
Methods & Clinical Development
Original Article

Hippocampal GAD67 Transduction Using rAAV8 Regulates Epileptogenesis in EL Mice

Kuniko Shimazaki,[1] Takashi Kobari,[1] Keiji Oguro,[1] Hidenori Yokota,[1] Yuko Kasahara,[2] Yoshiya Murashima,[3] Eiju Watanabe,[1] Kensuke Kawai,[1] and Takashi Okada[2]

[1]Department of Neurosurgery, Jichi Medical University, 3311-1 Yakushiji, Shimotsuke, Tochigi 329-0498, Japan; [2]Department of Biochemistry and Molecular Biology, Nippon Medical School, 1-1-5 Sendagi, Bunkyo-ku, Tokyo 113-8602, Japan; [3]Division of Human Health Sciences, Tokyo Metropolitan University, 7-2-10 Higashioku, Arakawa-ku, Tokyo, Japan

Gene therapy has been employed as a therapeutic approach for intractable focal epilepsies. Considering the potential of focal GABAergic neuromodulation in regulating epileptogenesis, the GABA-producing enzyme, γ-aminobutyric acid decarboxylase 67 (GAD67), is highly suitable for epilepsy therapy. The EL/Suz (EL) mouse is a model of multifactorial temporal lobe epilepsy. In the present study, we examined focal gene transduction in epileptic EL mice using recombinant adeno-associated virus serotype 8 (rAAV8) expressing human GAD67 to enhance GABA-mediated neural inhibition. Eight-week-old mice were bilaterally injected with rAAV8-GFP or rAAV8-GAD67 in the hippocampal CA3 region. After four weeks, the GAD67-transduced EL mice, but not the rAAV-GFP-treated EL mice, exhibited a significant reduction in seizure generation. The GAD67-mediated depression became stable after 14 weeks. The excitability of the CA3 region was markedly reduced in the GAD67-transduced EL mice, consistent with the results of the Ca^{2+} imaging using hippocampal slices. In addition, downregulation of c-Fos expression was observed in GAD67-transduced hippocampi. Our findings showed that rAAV8-GAD67 induced significant changes in the GABAergic system in the EL hippocampus. Thus, rAAV8-mediated GAD67 gene transfer is a promising therapeutic strategy for the treatment of epilepsies.

INTRODUCTION

Epilepsy is a common seizure disorder and represents one of the most prevalent neurological disorders worldwide.[1] Pharmacotherapy for epilepsy is merely symptomatic and thus does not provide curative therapeutic benefits. Despite advances in the development of antiepileptic drugs, refractory epilepsy remains a major clinical problem that affects up to 35% of patients with partial epilepsy.[2] For intractable cases in which patients develop tolerance and show pronounced side effects, surgical resection is the final option when a discrete seizure focus can be identified. Nonetheless, surgical intervention has palliative effects in many cases and can interfere with essential brain functions. Therefore, the use of the adeno-associated virus (AAV) vector to regulate epileptogenesis constitutes a major therapeutic advancement for the treatment of refractory epilepsy. In this context, intractable pharmacoresistant focal seizures are particularly suited for gene therapy. Effective and safe therapy becomes feasible by focal augmentation of endogenous anticonvulsive mechanisms without widespread side effects. In addition, direct application of antiepileptic proteins via direct delivery of gene therapy vectors can overcome therapeutic limitations because of the blood-brain barrier. Endogenous mechanisms for controlling neuronal excitability and epileptogenesis have emerged as new prospects for antiepileptogenic therapy.[2] However, systemic administration of antiepileptogenic compounds is compromised by limited bioavailability, poor penetration of the blood-brain barrier, and systemic distribution of the receptors. Therefore, local transduction strategies to facilitate therapeutic use of endogenous anticonvulsant principles are promising strategies when combined with surgical approaches. Augmentation of γ-aminobutyric acid (GABA)-mediated neural inhibition is one of the most effective antiepileptic mechanisms. Focal GABAergic neuromodulation exhibits good potential for regulating epileptogenesis. The GABA-producing enzyme, γ-aminobutyric acid decarboxylase (GAD), is highly suitable for expression in viral vectors given its established safety based on clinical gene therapy studies on Parkinson's disease.[3] The EL mouse is an inbred mutant strain that has been used as an animal model for generalized seizures[4] and a model of multifactorial temporal lobe epilepsy.[5] Epileptic seizures are mediated by two essential physiological mechanisms, namely, abnormality of cellular excitability and network hypersynchronization. The EL mouse is characterized by hippocampal disinhibition and has been suggested to exhibit decreased GABAergic synaptic transmission.[6]

AAV provides a highly promising platform for *in vivo* transduction because of its physical and chemical stability, broad host range (including non-proliferating neuromuscular tissues), and lack of pathogenicity. Transduction with vectors leads to the persistence of episomal forms in the infected tissue, safe and long-term transgene

Received 22 June 2018; accepted 29 December 2018;
https://doi.org/10.1016/j.omtm.2018.12.012.

Correspondence: Takashi Okada, Department of Biochemistry and Molecular Biology, Nippon Medical School, 1-1-5 Sendagi, Bunkyo-ku, Tokyo 113-8602, Japan.
E-mail: t-okada@nms.ac.jp
Correspondence: Kuniko Shimazaki, Department of Neurosurgery, Jichi Medical University, 3311-1 Yakushiji, Shimotsuke, Tochigi 329-0498, Japan.
E-mail: kunikos@jichi.ac.jp

 © 2019 The Author(s).
This is an open access article under the CC BY license (http://creativecommons.org/licenses/by/4.0/).

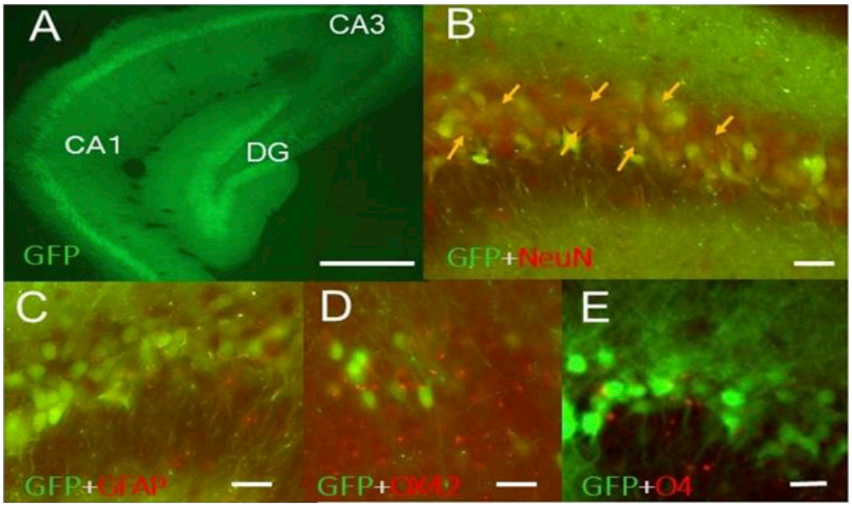

Figure 1. Transduction Profile of AAV8-GFP in the Hippocampus of EL Mouse

Fluorescence micrographs illustrating rAAV8-GFP-transduced cells (A), GFP positive cells (green) and NeuN (red :neuronal marker) positive cells (B), GFP positive cells (green) and GFP (red :astrocyte marker) positive cells (C), GFP positive cells (green) and OX42 (red :microglia marker) positive cells (D), and GFP positive cells (green) and O4 (red :oligodendryocyte marker) positive cells (E) in the ventral hippocampus. Arrows indicate typical colocalizing cells. CA1, field CA1 of Ammon's horn; CA3, field CA3 of Ammon's horn; DG, dentate gyrus. Scale bars, 500 μm for (A) and 50 μm for (B)–(E).

expression, and relatively low intrinsic immunogenicity.[7] Here, we investigated the effectiveness of a gene therapy approach using a recombinant adeno-associated virus serotype 8 (rAAV8) expressing the human GAD for the treatment of epileptogenesis in EL mice. Thus, rAAV8-mediated targeted GAD67 gene transfer serves as a promising therapeutic strategy for the treatment of epilepsies.

RESULTS

In Vivo Assessment of Transduction Profile of rAAV8-CMV-GFP

Initially, we assessed the extent of rAAV8 transduction and the distribution of the GFP transgene in the mouse brain. We evaluated the transduction pattern using rAAV8-GFP (control vector), which has the same backbone as rAAV8-GAD67. AAV8-GFP (1×10^{10} vector genome [v.g.]/μL) was infused into one side of the hippocampal CA3 region via stereotaxic injection (0.25 μL/min × 12 min: 3 μL of rAAV8). After 8 weeks of injection, we confirmed strong GFP gene expression throughout the hippocampus (Figure S1). Strong GFP signals were observed in the subiculum and dentate gyrus (DG). The results suggested rAAV8 can undergo both retrograde (CA3 to DG) and anterograde (CA3 to subiculum) transport. Many GFP-positive fibers and a few GFP-positive cells were observed in the contralateral hippocampus (Figure S1). The expression gradient of GFP was observed at a distance from the injection site. The number of GFP-positive cells in the olfactory bulb was less than that in the hippocampus. A few GFP-positive cells and fibers were observed in the ipsilateral side of olfactory bulb (more than 5 mm rostral to the hippocampal injection site). To assess the cellular expression pattern of rAAV8-GFP, double immunofluorescence was performed with GFP coupled with NeuN (neuron marker), GFAP (astrocyte marker), OX42 (microglia marker), and O4 (oligodendrocyte marker). Double-immunofluorescence analysis revealed that most GFP-expressing cells were NeuN positive (Figure 1).

rAAV8-GAD67 Prevents Seizures

The GAD67-expressing EL mice caused a significant decrease in the progression of seizures. rAAV8-GAD67 (bilaterally; n = 7) or rAAV8-GFP (bilaterally; n = 7) was infused bilaterally into the hippocampal CA3 regions of 8-week-old EL mice. The epileptic reaction after revolving stimulation was observed in both treatment groups every week. Figure 2 shows the seizure susceptibility that was assessed by our stimulation protocol. Change of seizure susceptibility of the rAAV8-GFP-treated mice and rAAV8-GAD67-treated mice was compared by Student's t test. The mice in the AAV8-GAD67-treated group showed significant inhibition of seizures. After four weeks (13 weeks of age), mice in the rAAV8-GAD67-infused group exhibited a significant reduction in seizure generation. The depression continued and reached stability from 14 weeks (23 weeks of age) after rAAV8-GAD67 injection. In naive EL mice, seizure susceptibility begins at 9 weeks of age and reached about 100% around 20 weeks of age (Figure S2). We observed no differences between naive and rAAV8-GFP-treated EL mice in terms of seizure susceptibility.

GAD Is Overexpressed after rAAV8-GAD67 Treatment in EL Hippocampi

GAD expression levels throughout the hippocampus are shown in the western blotting panel with duplicates (Figure 3). The antibody used for this experiment recognizes GAD65 as well as GAD67. A weak GAD band (65/67-kD) was visible in the naive hippocampal samples. Naive and vector-treated hippocampi showed differences in GAD expression. Results of western blots analysis suggested that the rAAV8-induced GAD67 expression increased over time. In 30-week-old groups, we observed a conspicuous difference between naive and rAAV8-GAD67-treated groups. Immunoblotting results showed the development of seizure resistance following rAAV8-GAD67 treatment.

Reduced $[Ca^{2+}]_i$ Elevation after OGD in AAV8-GAD67-Transduced EL Hippocampus

We observed an increase in intracellular Ca^{2+} ($[Ca^{2+}]_i$) in hippocampal slices after oxygen and glucose deprivation (OGD) (95% N_2/5% CO_2, glucose-free medium) using Rhod2-AM. (Figure 4). The first consequence of OGD is $[Ca^{2+}]_i$ accumulation following depolarization. Then, we examined the sequential changes in $[Ca^{2+}]_i$ following OGD of hippocampal slices from DDY mice (epileptic-free control,

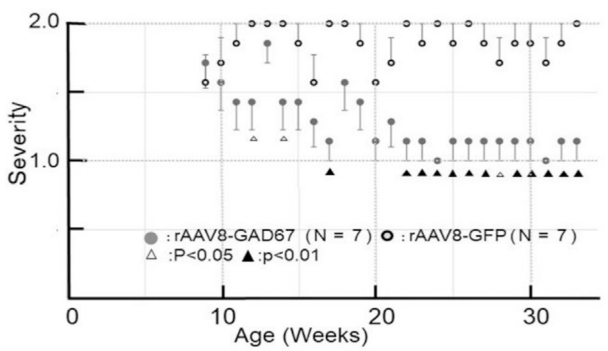

Figure 2. rAAV8-GAD67 Attenuates Seizure Sensitivity in EL Mice

Four weeks after delivery of rAAV8-GAD67, a remarkable reduction in the progression of seizures was observed. Severity was obtained by the following calculation: (2.0 × number of the mouse, which indicated score 2 + 1.0 × number of the mouse, which indicated score 1) ÷ total number of the mouse by each group. For instance, when 3 mice show tonic-clonic convulsion and 4 mice show head nodding or tail stiffening, epilepsy intensity can be calculated with 1.43: (2.0 × 3 + 1.0 × 4) ÷ 7 = 1.43.8, $p < 0.05$ relative to delivery of rAAV8-GAD67; 6, $p < 0.01$ relative to delivery of rAAV8-GAD67.

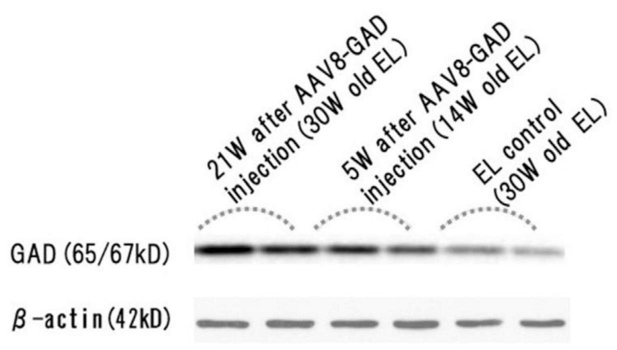

Figure 3. Western Blot Analysis of GAD65/67 Expression in the Hippocampi of Naive and Treated EL Mice

GAD65/67 expression levels in the EL hippocampus were examined by western blotting. GAD65/67 expression was upregulated in the hippocampi of rAAV8-GAD67-treated mice. Actin served as a loading control.

n = 10), naive EL mice (n = 10), and rAAV8-GAD67-treated EL mice (n = 7). The preceding results suggested that rAAV-GAD67 treatment decreased the excitability of the CA3 region. In the previous Ca^{2+} imaging studies, the temperature is influential to the time course of fluorescence change and the increase ratio. At a temperature of 32°C, fluorescence increased following a 10-min delay of perfusate change (artificial cerebro-spinal fluid [ACSF] to OGD) and reached maximum within 15 min after exposure to OGD. Changes in fluorescence were calculated at 15 min after exposing OGD relative to ACSF. The ratio (OGD 15 min/OGD 0 min) of fluorescence change was compared with rAAV8-GAD67-treated EL mice between naive EL mice and epilepsy-free DDY mice. In DDY mice, transient OGD led to increased $[Ca^{2+}]_i$ primarily in the CA1 region of the hippocampus. However, $[Ca^{2+}]_i$ in the CA1 and CA3 regions significantly increased after OGD in naive EL mice. In the rAAV8-GAD67-treated EL mice, $[Ca^{2+}]_i$ elevation in the CA3 region was inhibited significantly under the condition of OGD.

c-Fos Expression in the EL Hippocampus

The EL mice were sacrificed within 2 h after the final rotatory stimulus, and c-Fos expression was examined. Representative images of c-Fos expression in the hippocampus are presented in Figure S3. The c-Fos expression patterns in the naive EL mouse changed across the different developmental stages. c-Fos expression started with primary motor cortex, progressed to the locus coeruleus and prefrontal cortex, and appeared in the hippocampus and amygdala.[8] EL mice younger than 7 weeks did not undergo seizures, whereas 100% of 30-week-old mice underwent seizures. c-Fos expression level were compared between naive and rAAV8-GAD67-treated 30-week-old mice. At 30 weeks, a difference was observed between rAAV8-GAD67-treated mouse and naive mouse. Downregulation of c-Fos expression was observed in the CA1, CA3, and DG of rAAV8-GAD67-treated mice relative to those of naive mouse (Figures S3C and S3D).

DISCUSSION

AAV vectors often undergo long-distance anterograde and retrograde transport after brain injection, although the transduction varies widely among AAV serotypes.[7,9] To examine AAV8 transduction, 8-week-old mice were unilaterally injected with rAAV8-GFP in the hippocampal CA3 region and sacrificed at 8 weeks post-injection. As shown in Figure S1, local GFP expression was more pronounced in the hippocampus than in the ipsilateral cortex. Therefore, we considered reduction of epileptogenesis with local GAD67 expression using rAAV8.

Several reports showed weak expression of GABA and GAD in the parietal cortex and hippocampus in EL mice.[10,11] In the present study, we employed gene therapy to deliver GAD67 to the EL hippocampi. Focal gene transduction of the epileptic EL mice was examined, using rAAV8 expressing the human GAD67 to enhance GABA-mediated neural inhibition. The rAAV8-GAD67 injection led to significant inhibition of seizure progression. Results of the rhod-2 Ca^{2+} imaging study showed that excitability was markedly reduced in the GAD67-transduced hippocampi. Furthermore, downregulation of c-Fos expression in the hippocampal CA3 region was observed in parallel with acquisition of seizure resistance.

The hippocampi of EL mice propagated discharges that appeared to trigger manifest convulsions. In the mammalian brain, GABA biosynthesis depends on conversion of glutamate by GAD. GAD exists in two isoforms, GAD65 and GAD67, which are encoded by two distinct genes.[12] GAD65 expression was predominantly observed in the GABAergic synapse, while GAD67 was found to be evenly expressed throughout the cytosol.[13] Both isoforms synthesize GABA. Sloviter et al. were first to report that the source of GABA in mossy fiber terminals was GAD67. They showed that GABA and GAD67 were normally present in mossy fiber terminals of rat, monkey, and

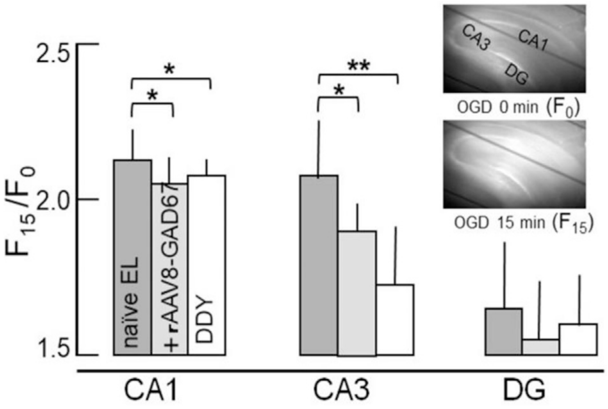

Figure 4. Fluorescence Quantitation in DDY, Naive EL, and rAAV8-GAD67-Treated EL Mice under Oxygen and Glucose Deprivation Exposure

The changes in $[Ca^{2+}]_i$ concentrations in the hippocampal slices after OGD exposure using rhod2-AM. Changing the incubation medium from normal to OGD caused, an abrupt increase in $[Ca^{2+}]_i$ in the CA1 subfield at 10 min after switching. The sequential changes in $[Ca^{2+}]_i$ following OGD in hippocampal slices from DDY, naive EL, and rAAV8-treated EL mice were examined using rhod2-AM. Regional differences in fluorescence signals increased after 15 min of exposure to ODG (inset panels; OGD 0 min and OGD 15 min). The hippocampal CA1, CA3, and DG regions were selected, and fluorescence signals were measured with an image processor. The baseline fluorescence (F_0) was normalized to 1.0. Values represent mean ± SD. Fluorescence intensities at time 15 (F_{15}/F_0, average ± SD) were 2.23 ± 0.12 (naive EL mice, n = 8), 2.09 ± 0.11 (rAAV8-GAD67-treated EL mice, n = 5), and 2.11 ± 0.05 (DDY mice, n = 8) in CA1; 2.11 ± 0.21 (naive EL mice), 1.83 ± 0.11 (rAAV8-GAD67-treated EL mice), and 1.71 ± 0.21 (DDY mice) in CA3; and 1.61 ± 0.26 (naive EL mice), 1.56 ± 0.21 (rAAV8-GAD67-treated EL mice), 1.58 ± 0.19 (DDY mice) in dentate gyrus (DG). The results were analyzed by Tukey-Kramer. *Risk rate is less than 5%. **Risk rate is less than 1%. Hippocampal slices from DDY and rAAV8-treated mice showed significantly lower $[Ca^{2+}]_i$ than those of naive EL mice after OGD exposure.

human hippocampus and that seizures upregulated GABA and both isoforms of GAD.[14] Furthermore, previous studies manifested that the GABA released from mossy fibers was synthesized from the 67-kDa isoform.[15–17] As shown in Figure 2, rAAV8-GAD67-treated and rAAV8-GFP-treated mice showed significant differences in the severity of epileptic seizure. rAAV8-GAD67 treatment significantly reduced epilepsy intensity in EL mice. In this experiment, the epilepsy-causing stimulus is 10 rotations. Treated EL mice had significantly lower total seizure scores but retained weak attacks. In the EL mice, seizure is induced by abrupt accelerating movements, including tossing the animal into the air or performing rotating movements.[18] In estimating the seizure score with a mild stimulus, such as tail suspension or tossing up, instead of rotating movements, there is a possibility that epilepsy was suppressed perfectly. As for tonic-clonic convulsions in GAD67-expressing mice, the frequency was reduced significantly, but significant decline in attack duration was not observed.

EL mice were found to exhibit disinhibition in the hippocampal CA3 region.[19] In naive EL mice, abrupt increases in the $[Ca^{2+}]_i$ concentration on the CA1 and CA3 subfields were observed under ischemic conditions. In the $[Ca^{2+}]_i$ elevation in the CA3 region was significantly depressed in the rAAV8-GAD67-treated EL mice. The preceding results suggested reducing CA3 excitability can inhibit epilepsy.

We examined the changes in c-Fos expression in the hippocampus by conducting immunohistochemical experiments. c-Fos immunoreactivity has been examined in several animal seizure models to identify brain regions involved in seizure activity. At 10 weeks of age, c-Fos expression emerged in the hippocampus and amygdala as mice approached the age of onset for seizure susceptibility. In naive EL mice, c-Fos expression started with the primary motor cortex at 3 weeks of age, progressed to the locus coeruleus and prefrontal cortex, and appeared in the hippocampus and amygdala.[8] Electrophysiological studies demonstrated that paroxysmal activities in the brains of naive EL mice arise from the parietal cortex and are augmented in the hippocampus.[4] The hippocampus does not act as the site origin but is eventually recruited to the seizure focus. The hippocampus is thought to be the source or primary mediator of seizure activity, and DG has been proposed as a control point for the spread of seizures.[20,21] c-Fos expression emerged in the hippocampus and amygdala by postnatal day (PND) 70, as mice approached the age of onset for seizure susceptibility.[16] The reduction in c-Fos expression in rAAV8-GAD67-treated EL hippocampi was consistent with reduced epileptogenesis.

Western blotting results indicated that the upregulation of GAD67 expression occurred in parallel with acquisition of epileptic resistance. The increase in GAD67 levels leads to GABA upregulation in the synaptic site. Therefore, GABA augmentation using rAAV8-mediated GAD67 transduction represents an effective strategy for seizure control. The rAAV8-mediated GAD67 transduction for enhancing GABA-mediated neural inhibition represents a good strategy for improving outcomes of the current therapeutic modalities, including anticonvulsive drugs and palliative surgery. Therefore, assessment of the rAAV8-GAD treatment for intractable cases is of great interest for increasing therapeutic benefits and safety.

Based on a different point of view, epilepsy results from an imbalance in the excitatory and inhibitory neurotransmitter systems. Thus, neuromodulators such as neuropeptide Y (NPY), adenosine, and galanin are thought to exert anticonvulsant and antiepileptogenic effects. NPY overexpression in the rat hippocampus induced by the rAAVs significantly reduced seizures in acute and chronic seizure models.[22,23] In the regulation of neuronal activity both under physiological conditions and during pathological hyperactivity accompanying seizures, important roles of NPY are emerging. However, the two major NPY receptors in the hippocampus, namely, the Y1 and Y2 receptors, appear to mediate opposite effects.[24] The Y2 receptor plays a critical role in mediating the anticonvulsive effect of NPY, whereas the Y1 receptor mediates the pro-convulsive effect of NPY. Therefore, long-term efficacy and safety in clinically relevant animal models remain to be improved. In the present study, epileptogenesis in EL mice was effectively regulated by rAAV8-mediated GAD67 transduction in the hippocampal CA3 region. The flow of

information within the hippocampus is largely unidirectional. Axons from the subiculum to project mainly to the DG. Some axons (mossy fibers) from DG project to the CA3 region, while fewer axons project to the CA1 region. The CA3 axons (Schaffer collaterals) loop up to the apical dendrites and then extend to the CA1 region. Axons from the CA1 region then project back to the entorhinal cortex.

The local transduction strategy developed here to augment GABA concentration would be useful for the treatment of intractable and pharmacoresistant focal seizures and for the development of seizure gene therapy strategies.

MATERIALS AND METHODS

Animals

Experimental animals included seizure-prone EL and control DDY mice, from which EL mice were originally derived but which are not prone to seizures. The EL mouse can be classified as a model of human temporal lobe epilepsy or complex partial seizures with secondary generalization.[25] The abnormally low distribution of GABA and low GAD activity in the parietal cortex and hippocampus were revealed by ultra-microenzymatic chemistry.[26] Male mice were used in this study. All mice were housed under light-dark cycle conditions maintained with 22°C to 24°C and 48% humidity. All mice were provided free access to food and water. Seizures in EL mice can occur spontaneously between 10 and 14 weeks after birth. Although the details of the onset of seizure susceptibility remain unclear,[4] the seizure threshold was lowered by repetition of the stimulation. In the experiments, mice were carefully exposed to routine rotating stimulation once a week starting at 4 weeks of age. All experiments were conducted in accordance with the protocols approved by the Institutional Animal Care and Use Committee of Jichi Medical University.

Proviral Plasmid Construction and Recombinant AAV Vector Production

We propagated the AAV8 vector proviral plasmid harboring GFP cDNA, the woodchuck hepatitis virus post-transcriptional regulatory element (WPRE), and the CAG promoter, a modified chicken actin promoter harboring a cytomegalovirus immediate early enhancer (CAG-EW). For total RNA extraction, mouse brains were homogenized a Multi-Beads Shocker (Yasui Kikai, Osaka, Japan), and RNA extraction was performed using the RNeasy Micro kit (QIAGEN, Crawley, UK). First-strand cDNA was synthesized using a Super Script III First-Strand Synthesis System for RT-PCR (Life Technologies, Carlsbad, CA, USA). The GAD-1-encoding cDNA fragment was used as a template for PCR amplification using PrimeSTAR HS DNA polymerase (Takara Bio, Shiga, Japan). The following PCR primer pairs were used: forward primer 5′-TCAATCGATTGAATTCCGCCACCATGGCA TCTTCCACTCCTTCGCCTGCAACC-3′ and reverse primer 5′-CC AAGCTTGCCTCGAGTTACAGATCCTGACCCAACCTCTCTAT CTC-3′. PCR produces subcloned into the EcoRI and *Xho* I site of pCMV, under the control of the cytomegalovirus (CMV) promoter, to generate pCMVmGAD-1. The expression cassette was further subcloned into the *Not* I site flanked by inverted terminal repeats (ITRs) in the AAV8 vector proviral plasmid. The vector genome was packaged into the pseudotyped AAV8 capsid in HEK293 cells. A large-scale cell culture method with an active gassing system was used for transfection.[27] The vector production process involved triple transfection of a proviral plasmid, a chimeric helper plasmid encoding AAV2 rep/AAV8 cap genes (pAAV2/8, a gift from Dr. James M. Wilson),[28–30] and an adenovirus helper plasmid pHelper (Stratagene). All viral particles were purified via CsCl gradient centrifugation, followed by a dual ion-exchange procedure with high-performance membrane adsorbers.[31] Viral titers were determined via qPCR, with real-time detection of PCR products using SYBR green and the MyiQ single-color detection system (Bio-Rad, Hercules, CA, USA). The following primer set was used for GFP: forward primer 5′-GTGAGCAAGG GCGAGGAG-3′ and reverse primer 5′-GTGGTGCAGA TGAACTT CAG G-3′ or the primer set targeting GAD-1 described earlier.

Stereotaxic Injection of rAAVs into Hippocampus

Male EL mice at 8 weeks of age were anesthetized with isoflurane (1% to 3%), and the intracerebral injection of AAV vectors was performed by stereotaxic surgery as previously described.[32] Mice were placed on a stereotaxic frame, and rAAV8-GAD (3 μL; 1×10^{10} v.g./μL; n = 7) or rAAV8-GFP (3 μL; 1×10^{10} v.g./μL; n = 7) was infused bilaterally into the CA3 region. Once the needle was in place, 3 μL of rAAV was infused at a rate of 0.25 μL/min for 12 min. After completing the injection (CA3 subfield: posterior 2.1 mm, lateral 2.6 mm, and dorso-ventral 1.4 mm), the needle was left in place for 5 min to allow the vector to diffuse from the injection site.

Immunohistochemistry

Mice were deeply anesthetized with pentobarbital sodium (Merck Sharp, Darmstadt, Germany) before intracardiac perfusion with saline, followed by treatment with 4% paraformaldehyde in 0.05 M PBS. Brain tissues were immediately removed, post-fixed overnight at 4°C, and soaked in a 15% sucrose solution in PBS at 4°C. Afterward, 40-μm-thick free-floating sections were prepared using a freezing microtome and subjected to histochemical analysis. For immunohistochemical analysis, brain sections were first incubated in a pretreatment buffer (0.1 M PBS, 0.1% Triton X-100, 1.5% goat blocking serum) for 0.5 h at room temperature. Then the sections were incubated with monoclonal antibodies targeting the following cellular markers: the neuronal marker NeuN (1:500; Chemicon MAB377); the astrocyte marker glial fibrillary acidic protein (1:1,000; Monosan PS032); the microglia marker OX42 (1:500; LsBio); and the oligodendrocyte marker O4 (1:200; Chemicon MAB345). Following overnight incubation, the sections were then incubated for 1 h with Alexa Fluor 546-conjugated goat-anti-rabbit immunoglobulin G (IgG) (1:1,000; Invitrogen) or Alexa Fluor 546-conjugated goat-anti-mouse IgG (1:1,000; Invitrogen) in PBS at room temperature. GFP antibody was chosen from the mouse origin (1:500; GeneTex GNT628528) and the rabbit origin (1:1,000; Abcam ab6556). For c-Fos detection, sections were incubated with rabbit anti-c-Fos polyclonal antibody (1:1,000, Calbiochem PC05) in pretreatment buffer overnight at 4°C. On the second day, brain sections

were incubated with Alexa Fluor 488-conjugated goat-anti-rabbit IgG (1:1,000; Invitrogen) for 1 h at room temperature.

Scoring of Epileptic Symptom

Mice were suspended by the tail in home cages about 30 to 40 cm above the bedding, turned ten times (about 10 s), and subsequently placed in clean cages with fresh chip bedding. The convulsant responses of the EL mice were observed after rotatory stimulation and were scored based on the following three points (0 to 2). Score 0: no behavioral signals of seizure (mice that exhibited vocalization and twitching without the characteristics of behavioral signs were not considered to show seizure activity). Score 1: rigidity with tail raising, rigidity with tail stiffing, head nodding, facial or forelimb clonus, wild running (single or mixed symptom). Score 2: generalized tonic-clonic seizure (disturbance in posture). The reaction of each mouse was video recorded.

Western Blotting

Mice were anesthetized with ether before decapitation for western blotting. The hippocampi were dissected and stored at −80°C until protein analysis. Samples were then sonicated in ten volumes of SDS sample buffer and centrifuged at 15,000 rpm for 15 min at 4°C, followed by 3 min of incubation at 100°C. Aliquots containing 50 μg of protein were subjected to 10.0% acrylamide gel electrophoresis following standard procedures. Western blotting was performed on polyvinylidene fluoride (PVDF) membranes using a semi-dry blotting apparatus (HorizeBLOT 4M-R, ATTO, Japan). Immunodetection was performed using a GAD67 antibody (1/500 dilution; OriGene TA308881). After overnight incubation at 4°C with the primary antibody, the membranes were incubated in alkaline phosphatase-conjugated secondary antibody (1/1,000 to 1/2,000 dilution; Promega, Madison, WI, USA) at room temperature for 1 h. Immunoreactive bands were visualized using 5-bromo-4-chloro-3-indolyl phosphate (Sigma-Aldrich) and nitro blue tetrazolium (Sigma-Aldrich) in 100 mM Tris/HCl (pH 9.5) containing 100 mM NaCl and 5 mM $MgCl_2$. The membranes were rinsed for 15 min between each step with Tris/HCl buffer (pH 7.4) containing 0.1% Tween 20.

$[Ca^{2+}]_i$ Imaging

Brain tissues were immersed in ice-cold ACSF solution, consisting of 124 mM NaCl, 1.5 mM KCl, 2.5 mM $CaCl_2$, 1 mM $MgCl_2$, 1.25 mM NaH_2PO_4, 22 mM $NaHCO_3$, and 10 mM glucose. Brain slices (350 μm) were incubated in the ACSF (pH 7.3) equilibrated with 95% O_2 to 5% CO_2 for 30 min at room temperature and loaded with a fluorescent Ca^{2+} indicator, 10 mM rhod2-AM (Dojindo, Kumamoto, Japan), for 90 min. Rhod2-AM-loaded slices were placed in a small flow-through chamber on a microscope stage and continuously perfused (2 mL/min) with the equilibrated ACSF solution at 32°C . Fluorescence images of rhod-2-loaded slices (>580 nm), at the excitation wavelength of 520–550 nm were obtained through a low-magnification objective lens (UM Plan Fl 10×, Olympus, Tokyo, Japan). Data were recorded using the imaging system as described previously with modifications.[33] Images were sequentially captured every 20 s. For quantitative analysis, the fluorescence intensity at each time point was divided by that of the initial image stored as a reference.

Statistical Analysis

To analyze the seizure susceptibility of the AAV8 vector-treated group, Student's t test was used. Statistical significance was set at $p < 0.05$. $[Ca^{2+}]_i$ changes after ischemic conditions were analyzed by the Turkey-Kramer method (risk rate is less than 5% and risk rate is less than 1% considering as the significant level). Data were presented as the mean and SD throughout the figures.

SUPPLEMENTAL INFORMATION

Supplemental Information includes three figures and can be found with this article online at https://doi.org/10.1016/j.omtm.2018.12.012.

AUTHOR CONTRIBUTIONS

K.S., K.K., and T.O. designed the experiments and wrote the manuscript. T.K., K.O., and H.Y. performed animal studies. Y.K. generated rAAV. Y.M. and E.W. provided the EL mice.

ACKNOWLEDGMENTS

We thank Dr. James M. Wilson for providing the helper plasmid pAAV2/8. This work was supported by Grants in Aid for Scientific Research from the Ministry of Education, Culture, Sports, Science and Technology of Japan (MEXT, 26293328 and 25460501). We thank Dr. Kazumi Motoki, Ms. Kazue Kinoshita, and Ms. Yumiko Itou for their technical support.

REFERENCES

1. Goldberg, E.M., and Coulter, D.A. (2013). Mechanisms of epileptogenesis: a convergence on neural circuit dysfunction. Nat. Rev. Neurosci. *14*, 337–349.
2. Boison, D. (2007). Cell and gene therapies for refractory epilepsy. Curr. Neuropharmacol. *5*, 115–125.
3. Kaplitt, M.G., Feigin, A., Tang, C., Fitzsimons, H.L., Mattis, P., Lawlor, P.A., Bland, R.J., Young, D., Strybing, K., Eidelberg, D., and During, M.J. (2007). Safety and tolerability of gene therapy with an adeno-associated virus (AAV) borne GAD gene for Parkinson's disease: an open label, phase I trial. Lancet *369*, 2097–2105.
4. Suzuki, J. (2013). Neuronal mechanism of epileptogenesis in EL mouse. Proc. Jpn. Acad., Ser. B, Phys. Biol. Sci. *89*, 270–280.
5. Heinrichs, S.C., and Seyfried, T.N. (2006). Behavioral seizure correlates in animal models of epilepsy: a road map for assay selection, data interpretation, and the search for causal mechanisms. Epilepsy Behav. *8*, 5–38.
6. Ono, T., Fueta, Y., Janjua, N.A., Ohmori, O., Ohno, K., Murai, Y., and Mita, T. (1997). Granule cell disinhibition in dentate gyrus of genetically seizure susceptible El mice. Brain Res. *745*, 165–172.
7. Aschauer, D.F., Kreuz, S., and Rumpel, S. (2013). Analysis of transduction efficiency, tropism and axonal transport of AAV serotypes 1, 2, 5, 6, 8 and 9 in the mouse brain. PLoS ONE *8*, e76310.
8. Leussis, M.P., and Heinrichs, S.C. (2007). Temporal ontogeny of circuit activation prior to the onset of seizure susceptibility in EL/Suz mice. Neuroscience *145*, 33–41.
9. Castle, M.J., Gershenson, Z.T., Giles, A.R., Holzbaur, E.L., and Wolfe, J.H. (2014). Adeno-associated virus serotypes 1, 8, and 9 share conserved mechanisms for anterograde and retrograde axonal transport. Hum. Gene Ther. *25*, 705–720.
10. Murashima, Y.L., and Suzuki, J. (1989). The GABAergic role of the epileptogenesis in the El mouse. Jpn. J. Psychiatry Neurol. *43*, 557–558.

11. Fueta, Y., Kawano, H., Ono, T., Mita, T., Fukata, K., and Ohno, K. (1998). Regional differences in hippocampal excitability manifested by paired-pulse stimulation of genetically epileptic El mice. Brain Res. *779*, 324–328.

12. Bu, D.F., Erlander, M.G., Hitz, B.C., Tillakaratne, N.J., Kaufman, D.L., Wagner-McPherson, C.B., Evans, G.A., and Tobin, A.J. (1992). Two human glutamate decarboxylases, 65-kDa GAD and 67-kDa GAD, are each encoded by a single gene. Proc. Natl. Acad. Sci. USA *89*, 2115–2119.

13. Kaufman, D.L., Houser, C.R., and Tobin, A.J. (1991). Two forms of the gamma-aminobutyric acid synthetic enzyme glutamate decarboxylase have distinct intraneuronal distributions and cofactor interactions. J. Neurochem. *56*, 720–723.

14. Sloviter, R.S., Dichter, M.A., Rachinsky, T.L., Dean, E., Goodman, J.H., Sollas, A.L., and Martin, D.L. (1996). Basal expression and induction of glutamate decarboxylase and GABA in excitatory granule cells of the rat and monkey hippocampal dentate gyrus. J. Comp. Neurol. *373*, 593–618.

15. Maqueda, J., Ramírez, M., Lamas, M., and Gutiérrez, R. (2003). Glutamic acid decarboxylase (GAD)67, but not GAD65, is constitutively expressed during development and transiently overexpressed by activity in the granule cells of the rat. Neurosci. Lett. *353*, 69–71.

16. Ramírez, M., and Gutiérrez, R. (2001). Activity-dependent expression of GAD67 in the granule cells of the rat hippocampus. Brain Res. *917*, 139–146.

17. Schwarzer, C., and Sperk, G. (1995). Hippocampal granule cells express glutamic acid decarboxylase-67 after limbic seizures in the rat. Neuroscience *69*, 705–709.

18. Fueta, Y., Mita, T., and Matsuoka, S. (1983). Experimental animal epilepsy. A new device for the induction of epileptic seizures in the murine, El mouse. J. UOEH *5*, 359–364.

19. Suzuki, J. (2004). Investigations of epilepsy with a mutant animal (EL mouse) model. Epilepsia *45* (*Suppl 8*), 2–5.

20. Lothman, E.W., Bertram, E.H., 3rd, and Stringer, J.L. (1991). Functional anatomy of hippocampal seizures. Prog. Neurobiol. *37*, 1–82.

21. Lothman, E.W., Stringer, J.L., and Bertram, E.H. (1992). The dentate gyrus as a control point for seizures in the hippocampus and beyond. Epilepsy Res. Suppl. *7*, 301–313.

22. Powell, K.L., Fitzgerald, X., Shallue, C., Jovanovska, V., Klugmann, M., Von Jonquieres, G., O'Brien, T.J., and Morris, M.J. (2018). Gene therapy mediated seizure suppression in genetic generalised epilepsy: neuropeptide Y overexpression in a rat model. Neurobiol. Dis. *113*, 23–32.

23. Noe, F., Vaghi, V., Balducci, C., Fitzsimons, H., Bland, R., Zardoni, D., Sperk, G., Carli, M., During, M.J., and Vezzani, A. (2010). Anticonvulsant effects and behavioural outcomes of rAAV serotype 1 vector-mediated neuropeptide Y overexpression in rat hippocampus. Gene Ther. *17*, 643–652.

24. Lin, E.J., Young, D., Baer, K., Herzog, H., and During, M.J. (2006). Differential actions of NPY on seizure modulation via Y1 and Y2 receptors: evidence from receptor knockout mice. Epilepsia *47*, 773–780.

25. Seyfried, T.N., and Glaser, G.H. (1985). A review of mouse mutants as genetic models of epilepsy. Epilepsia *26*, 143–150.

26. Murashima, Y.L., Kassamo, K., and Suzuki, J. (1996). Developmental and seizure-related regional differences in immediate early gene expression and GABAergic abnormalities in the brain of EL mice. Epilepsy Res. *26*, 3–14.

27. Okada, T., Nomoto, T., Yoshioka, T., Nonaka-Sarukawa, M., Ito, T., Ogura, T., Iwata-Okada, M., Uchibori, R., Shimazaki, K., Mizukami, H., et al. (2005). Large-scale production of recombinant viruses by use of a large culture vessel with active gassing. Hum. Gene Ther. *16*, 1212–1218.

28. Lin, J., Zhi, Y., Mays, L., and Wilson, J.M. (2007). Vaccines based on novel adeno-associated virus vectors elicit aberrant CD8+ T-cell responses in mice. J. Virol. *81*, 11840–11849.

29. Gao, G.P., Alvira, M.R., Wang, L., Calcedo, R., Johnston, J., and Wilson, J.M. (2002). Novel adeno-associated viruses from rhesus monkeys as vectors for human gene therapy. Proc. Natl. Acad. Sci. USA *99*, 11854–11859.

30. Gao, G., Vandenberghe, L.H., Alvira, M.R., Lu, Y., Calcedo, R., Zhou, X., and Wilson, J.M. (2004). Clades of adeno-associated viruses are widely disseminated in human tissues. J. Virol. *78*, 6381–6388.

31. Okada, T., Nonaka-Sarukawa, M., Uchibori, R., Kinoshita, K., Hayashita-Kinoh, H., Nitahara-Kasahara, Y., Takeda, S., and Ozawa, K. (2009). Scalable purification of adeno-associated virus serotype 1 (AAV1) and AAV8 vectors, using dual ion-exchange adsorptive membranes. Hum. Gene Ther. *20*, 1013–1021.

32. Okada, T., Shimazaki, K., Nomoto, T., Matsushita, T., Mizukami, H., Urabe, M., Hanazono, Y., Kume, A., Tobita, K., Ozawa, K., and Kawai, N. (2002). Adeno-associated viral vector-mediated gene therapy of ischemia-induced neuronal death. Methods Enzymol. *346*, 378–393.

33. Shimazaki, K., Nakamura, T., Nakamura, K., Oguro, K., Masuzawa, T., Kudo, Y., and Kawai, N. (1998). Reduced calcium elevation in hippocampal CA1 neurons of ischemia-tolerant gerbils. Neuroreport *9*, 1875–1878.

OMTM, Volume *13*

Supplemental Information

Hippocampal GAD67 Transduction Using rAAV8 Regulates Epileptogenesis in EL Mice

Kuniko Shimazaki, Takashi Kobari, Keiji Oguro, Hidenori Yokota, Yuko Kasahara, Yoshiya Murashima, Eiju Watanabe, Kensuke Kawai, and Takashi Okada

Supplemental Figures and Legends

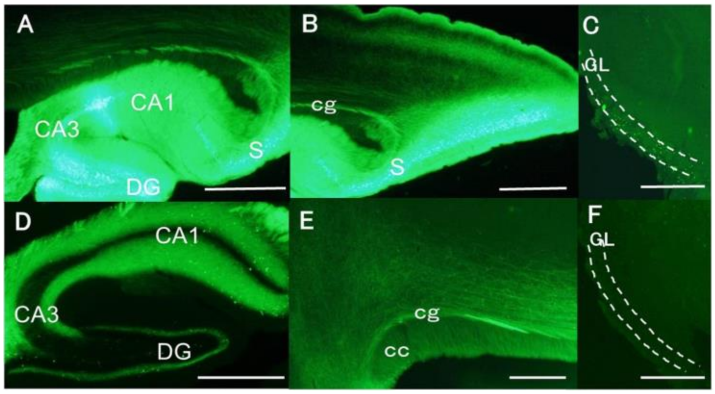

Figure S1. Anterograde and Retrograde Transduction of rAAV8-GFP.
Representative micrographs of 40-µm sagittal sections were prepared. After 8 weeks of rAAV8-GFP treatment, strong GFP expression was observed in the injection site of hippocampus (A). In the contralateral site, many GFP-positive fibers and a few GFP-positive cells were observed (D, E). A little GFP expression was seen in the ipsilateral site of olfactory bulb (C). S, subiculum; CA1, field CA1 of Ammon's horn; CA3, field CA3 of Ammon's horn; DG, dentate gyrus; cc, corpus callosum; GL, glomerular layer; cg; cingulum. Bars = 500 µm for whole sections.

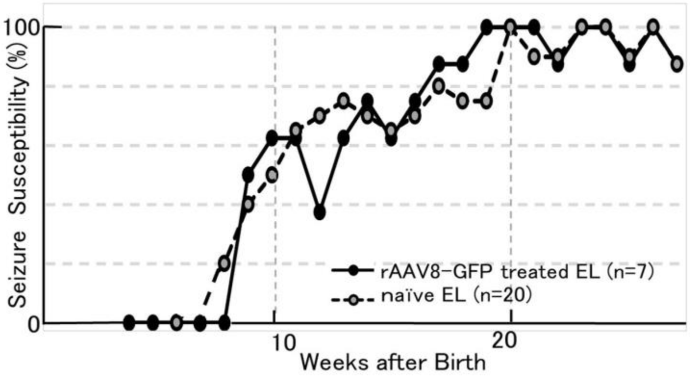

Figure S2. Development and Seizure Susceptibility of EL Mice
The seizure susceptibility increases with growth. In naïve EL mice, seizure susceptibility begins 9 weeks of age and reached about 100% around 20 weeks of age. There is no difference between naïve and rAAV8-GFP treated mice. In addition to tonic-clonic convulsion, behavioral signals of seizure activity in EL mice could include tail raising, head nodding, tail stiffening, facial/forelimb clonus and wild running. Mice that exhibited vocalization and twitching without the characteristics of behavioral signs were not considered seizure activity. A tonic-clonic convulsion is seen around 12 weeks of age.

Figure S3

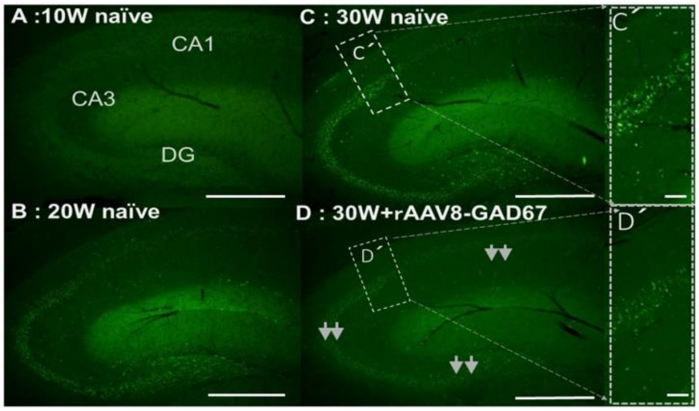

Figure S3. Representative Images of c-Fos Immunostaining in the Hippocampus.
The mice were sacrificed within 2 h after the final stimulus. For histochemical analysis, five mouse brains were fixed, and sagittal sections were prepared. Increased staining was clearly visible in the hippocampus of naïve EL mice. In 20-weeks-old naïve EL mice, c-Fos expression was upregulated in the CA3 region and DG (B). Moreover, the c-Fos expression was upregulated in the CA3 region in 30-week-old naïve EL mice (C and C´). At 21 weeks post-injection c-Fos expression levels were downregulated in 30-week-old EL mice compared to those of naïve EL mice (D and D´). Arrows indicate downregulated c-Fos expression. Scale bars indicate 250 µm and 100 µm. Double arrows indicate areas with reduced c-Fos expression.
